# SCOPE1: a randomised phase II/III multicentre clinical trial of definitive chemoradiation, with or without cetuximab, in carcinoma of the oesophagus

**DOI:** 10.1186/1471-2407-11-466

**Published:** 2011-10-28

**Authors:** Christopher N Hurt, Lisette S Nixon, Gareth O Griffiths, Ruby Al-Mokhtar, Simon Gollins, John N Staffurth, Ceri J Phillips, Jane M Blazeby, Tom D Crosby

**Affiliations:** 1 Wales Cancer Trials Unit, School of Medicine, Cardiff University, Cardiff, UK; 2 Cardiff NCRI RTTQA group, Department of Medical Physics, Velindre Cancer Centre, Cardiff, UK; 3 Department of Clinical Oncology, North Wales Cancer Treatment Centre, Rhyl, UK; 4 Institute of Cancer and Genetics, School of Medicine, Cardiff University, Cardiff, UK; 5 Swansea Centre for Health Economics, College of Human and Health Sciences, Swansea University, Swansea, UK; 6 Surgical Research Unit, School of Social and Community Medicine, University of Bristol, Bristol, UK; 7 Velindre Cancer Centre, Cardiff, UK

## Abstract

**Background:**

Chemoradiotherapy is the standard of care for patients with oesophageal cancer unsuitable for surgery due to the presence of co-morbidity or extent of disease, and is a standard treatment option for patients with squamous cell carcinoma of the oesophagus. Modern regimens of chemoradiotherapy can lead to significant long-term survival. However the majority of patients will die of their disease, most commonly with local progression/recurrence of their tumours. Cetuximab may overcome one of the principal mechanisms of tumour radio-resistance, namely tumour repopulation, in patients treated with chemoradiotherapy.

The purpose of this research is first to determine whether the addition of cetuximab to definitive chemoradiotherapy for treatment of patients with non-metastatic carcinoma of the oesophagus is active (in terms of failure-free rate), safe, and feasible within the context of a multi-centre randomised controlled trial in the UK. If the first stage is successful then the trial will continue to accrue sufficient patients to establish whether the addition of cetuximab to the standard treatment improves overall survival.

**Methods/Design:**

SCOPE1 is a two arm, open, randomised multicentre Phase II/III trial. Eligible patients will have histologically confirmed carcinoma of the oesophagus and have been chosen to receive definitive chemoradiotherapy by an accredited multidisciplinary team including a specialist Upper GI surgeon. 420 patients will be randomised to receive definitive chemoradiotherapy with or without cetuximab using a 1:1 allocation ratio.

During Phase II of the study, the trial will assess safety (toxicity), activity (failure-free rate) and feasibility (recruitment rate and protocol dose modifications/delays) in 90 patients in the experimental arm. If the experimental arm is found to be active, safe, and feasible by the Independent Data Monitoring Committee then recruitment will continue into Phase III. This second stage will recruit a further 120 patients into each arm and compare the overall survival of both groups.

All patients randomised into Phase II will contribute to the Phase III comparison of overall survival. In addition to overall survival, Phase III of the study will also assess toxicity, health related quality of life and cost effectiveness. A detailed radiotherapy protocol and quality assurance procedure has been incorporated into this trial.

**Trial registration:**

ISRCTN: ISRCTN47718479

## Background

Worldwide, oesophageal cancer is the eighth most common cancer, responsible for an estimated 482,300 new cases and 406,800 deaths in 2008 and is the fifth highest in mortality rate among tumour sites [1]. In the UK, there were 7,966 new cases of oesophageal cancer diagnosed in 2007 and it is responsible for approximately 4% of all cancer deaths with over 7,600 people dying in 2008 [2]. There are two main histological types of oesophageal cancer, squamous cell carcinoma (SCC) and adenocarcinoma (AC). Recently there has been an increase in the numbers of adenocarcinomas of the lower oesophagus and gastro-oesophageal junction in populations of the western world [1] whilst the incidence of squamous carcinoma has fallen slightly. In the UK, there has been a 60% increase in oesophageal carcinoma incidence in males over the past 30 years [2].

Surgery has long been, and remains, the cornerstone for cure of oesophageal cancer and is considered for all patients with potentially resectable oesophageal cancer who are fit for surgery and have no evidence of distant disease. Approximately 23% of patients survive 5 years with the most commonly used surgical treatment strategy [3]. However, rates of surgical intervention in the UK are as low as 20% of all cases[4]. The most recent figures (for patients diagnosed between 2001 and 2006) show that the 5-year survival of all patients with oesophageal cancer is 10 per cent [4].

### Chemoradiotherapy (CRT) in oesophageal cancer

In a pivotal study [5], US Intergroup RTOG-8501 randomised 121 patients with squamous cell carcinoma (SCC) or adenocarcinoma (AC) to receive CRT (4 cycles cisplatin and 5-Fluorouracil (5FU), the first 2 cycles given concurrently with 50Gy radiotherapy in 25 fractions) or radiotherapy alone (64Gy in 32 fractions). This trial, together with a subsequent systematic review [6], demonstrated a survival superiority of CRT over radiotherapy alone (1-year mortality odds ratio 0.61, 95% CI 0.31-0.89, P < 0.001), albeit at the expense of increased toxicity. This and other reported studies [5,7-12] have been predominantly in patients with SCC and have demonstrated a remarkably consistent median survival of 14-18 months and 2 year overall survival of 30-40% with CRT.

In the UK, most experience has been gained in those patients, both with SCC and AC, deemed unsuitable for surgery due to the presence of co-morbidity or extent of disease [8,13]. Despite the expected poor prognosis of this patient group, of the 266 patients who were deemed inoperable at one UK centre between 1995 and 2009, the median survival was 20.6 months (2 and 5 year survival 44% (95% CI: 37, 50%) and 20% (95% CI: 14, 26%) respectively) [8]. In this study 42% of patients suffered grade 3 and 7% grade 4 toxicities, mainly mucosal and haematological due to the chemotherapy.

### Rational for standard chemotherapy agents

Concurrent CRT regimens have been based upon cisplatin and 5FU. Both have good single agent activity in oesophageal malignant disease and are amongst the best radio-sensitisers in tumour models [14,15]. The regimen used most frequently in the UK involves conformal external beam radiotherapy, 50Gy in 25 fractions over 5 weeks, with 2 cycles cisplatin and 5FU given concurrently, with or without a further 2 cycles of the same chemotherapy, given as a neo-adjuvant phase [16]. The latter, as well as delivering additional systemic therapy, allows time for careful radiotherapy planning, frequently improves the patients' dysphagia and 'debulks' the tumour prior to radiotherapy.

5FU has historically been given as a continuous infusion throughout treatment. Capecitabine (Xeloda™), an oral fluoropyrimidine, sequentially converted to 5FU via 3 enzymes located in liver and tumour tissue, mimics the effect of continuous infusional 5FU. Capecitabine has been shown to be at least as effective as infusional 5FU in advanced oesophago-gastric cancer [17] and the use of capecitabine instead of infusional 5FU during Upper GI concurrent CRT has now become standard practice in some centres e.g. Velindre Cancer Centre, Cardiff and the Royal Marsden Hospital, Sutton. Capecitabine is also being used concurrently with radiotherapy to treat other Upper GI tumour sites [18]. In patients with significant dysphagia capecitabine can be dissolved in warm water and swallowed or even administered via a naso-gastric tube.

### Cetuximab and anti-EGFR therapies

The majority of patients who relapse do so within the previously irradiated area [5,10,19]. The reported local failure rate in recent studies is 45-58% of patients treated with CRT. This may reflect the advanced nature of the disease, however factors such as tumour cell repopulation during radiation therapy have long been known to be an important mechanism of radio-resistance [20,21]. Radiotherapy stimulates tumour cell growth through activation of the EGF receptor complex causing homodimerisation of the extracellular receptor inducing autophosphorylation of the intracellular tyrosine kinase (TK) domain [22], in turn stimulating a number of intracellular signal transduction pathways such as the ras-raf-MAPK pathway [21]. This activation sequence can be blocked by the monoclonal antibody, cetuximab, preventing radiotherapy induced growth stimulation.

Bonner et al [23] reported results of a trial in patients with locally advanced squamous cell carcinoma of the head and neck, a disease with many similarities to oesophageal cancer, which tested the benefits of the addition of cetuximab to radiotherapy. 424 patients were randomised to receive 70-76.8Gy of radiotherapy with or without cetuximab. Patients were followed up for a minimum of 2 years. The addition of the antibody was well tolerated, notably without an increase in mucositis, although 34% developed predictable and mostly manageable G3-4 acute skin reactions (compared to 18%). With the addition of cetuximab there was improved local control (47% vs 34% at 3 year, P < 0.01) and overall survival (55% vs 45% at 3 years, p = 0.05) with nearly a doubling in median survival (49 vs 29 months). More recently, a phase II study combining adjuvant cetuximab and CRT after surgery in patients with head and neck cancer has shown the addition of cetuximab to be feasible and tolerated with predictable toxicity [24].

In patients with metastatic colorectal cancer, cetuximab has shown significant activity in combination with chemotherapy in patients who have relapsed on the same chemotherapy regimen given alone [25]. EXPERT-C, a randomised phase II study comparing neoadjuvant chemotherapy before CRT and TME with and without cetuximab in patients with MRI selected high-risk operable rectal adenocarcinoma, has shown significant improvement in 3 year OS with the addition of cetuximab (81% vs 96%; HR 0.27, p = 0.035) in the KRAS + BRAF wild type population although in the all treated population there was no difference [26]. Skin toxicity was increased with cetuximab during neoadjuvant chemotherapy and CRT and diarrhoea was increased during CRT. XERXES, a multicentre pilot trial to examine the role of cetuximab when added to a schedule of capecitabine plus pelvic radiation in patients who have locally advanced primary rectal cancers, is still recruiting.

Anti-EGFR therapies have been combined with CRT in the treatment of thoracic malignancies. 15 patients with stage III non-small cell lung cancer received gefitinib with 60Gy radiotherapy, given concurrently with carboplatin and escalating doses of paclitaxel. This anti-EGFR small molecular agent was well tolerated even in the cohort receiving full dose chemotherapy, with an impressive response rate of 91% [27]. RTOG 0324, a Phase II study of primary CRT and cetuximab in Stage IIIA/B non-small cell lung cancer, enrolled 93 patients of whom 87 were evaluable [28]. Response rate was 62%, median survival was 22.7 months, and 24-month overall survival was 49.3%. Adverse events related to treatment included 20% grade 4 hematologic toxicities, 8% grade 3 esophagitis, and 7% grade 3 to 4 pneumonitis. It concluded that the combination of cetuximab with CRT is feasible and shows promising activity.

In oesophageal cancer, cetuximab has been tested in combination with irinotecan, cisplatin and concurrent radiotherapy in Phase II studies: a single centre study (NCT00165490, 02-012) and in the SWOG-SO414 study. In the former study, in resectable tumours, 17 of 39 planned patients had completed the protocol by the ASCO 2006 meeting [29]. Grade 3/4 toxicities seen were diarrhoea (9 patients), neutropenia (9 patients), febrile neutropenia (5 patients), anorexia (5 patients), vomiting (4 patients), fatigue (3 patients) and mucositis (1 patients). The study is still in follow up. The SWOG-S0414 study, in unresectable tumours, closed due to slow accrual after recruiting 21 patients. 48% and 29% of patients had Grade 3 and 4 toxicity respectively [30].

In a larger series, 37 patients with oesophago-gastric cancer underwent carboplatin AUC = 2, paclitaxel 50 mg/m2, cetuximab 250 mg/m2 weekly concurrently with 50.4Gy radiotherapy. There were no Grade 4 non-haematological toxicities, 20% of patients had Grade 3 oesophagitis. Eighteen of 27 (67%) had no residual disease on endoscopic biopsy after treatment and 7 of 16 (43%) who underwent surgery had a complete pathological response [31]. Building on their pivotal work with cetuximab and radiotherapy in head and neck cancer, Dobelbower et al have performed a Phase 1 study of cisplatin, infusional 5FU, erlotinib (anti-EGFR small molecule) with 50.4Gy radiotherapy in 11 patients with oesophageal cancer. There were no unexpected toxicities [32].

Since the start of the SCOPE1 trial, a phase II study of neoadjuvant CRT with oxaliplatin and infusional 5-FU plus cetuximab followed by postoperative docetaxel and cetuximab has completed in patients with operable adenocarcinoma of the oesophagus [33]. It was stopped after 22 patients had been recruited due to unacceptable toxicity (4 post operative deaths all from ARDS) although it showed promising activity based on pathological complete response rate (pCR) rate (7 patients). However, a Phase II study of CRT with FOLFOX plus cetuximab in 79 patients with locally advanced cardia or oesophageal cancer has also completed recently and concluded that it is active (overall response rate after CRT was 77.2%) and has an acceptable toxicity profile in patients with locally advanced cardia or oesophageal cancer [34].

Other Phase I/II studies of cetuximab in oesophageal cancer are still ongoing: EXCEL (NCT00815308, 44 patients in China), FFCD 0505 (NCT00544362, 45 patients in France), and NCT00425425 (43 patients in Germany). RTOG 0436, a Phase III trial (420 patients) evaluating the addition of cetuximab to paclitaxel, cisplatin, and radiation for patients with oesophageal cancer who are treated without surgery, is still in recruitment. A Swiss Phase III trial (NCT01107639, 300 patients) giving CRT with or without cetuximab followed by surgery is also in recruitment.

### Endpoint assessment

Assessing response to CRT, without surgically removing the oesophagus for histological examination, is notoriously difficult. Endoscopy negative rates following CRT range from 30-80% (mean 61%) [35-42]. Although not a reliable marker for pCR within the surgically resected specimen [38,42] endoscopic response is strongly correlated with long-term survival [35-37]. No other technique has been shown to be more reliable though trials are ongoing into the role of 18-FDG PET and EUS guided FNA in this setting. PET assesses metabolic activity (as opposed to size) so is able to determine benign from malignant lesions and is not only effective in detecting the primary tumour, but also is more sensitive in detecting small nodal and distant metastases [43]. It is likely to become more commonly used after the results of larger trials and availability of equipment. It is not clear whether the presence of the EGF receptor predicts for response to cetuximab. Although not a pre-requisite for entry into this study, this and other predictors of response will be the subject of associated translational research.

### Main research question

The main study aim of the SCOPE1 trial is to assess the effect on overall survival of adding cetuximab to the standard treatment. We will also examine the effect on toxicity, health related quality of life (HRQL), and cost effectiveness. In this paper we describe the study protocol.

## Methods/Design

### Study Design

**Figure 1:**
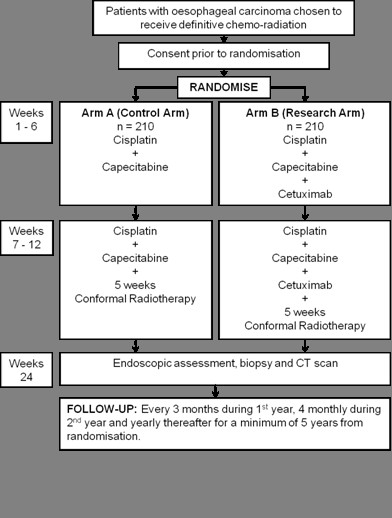
**Trial Schema**.

SCOPE1 is a two arm, open, randomised Phase II/III trial. It is being run in approximately 50 participating centres throughout the UK. Eligible patients will have histologically confirmed carcinoma of the oesophagus (squamous cell, adenocarcinoma, or undifferentiated carcinoma) and have been chosen to receive definitive CRT by an accredited multidisciplinary team (MDT) including a specialist Upper GI surgeon. At randomisation, participants are assigned to either the control arm or the research arm using a 1:1 allocation ratio (see Figure 1). The control treatment consists of cisplatin (60 mg/m2 IV Day 1 of 21 day cycle for 4 cycles) and capecitabine (625 mg/m2 po bd Days 1-84) and, from week 7, radiotherapy (50Gy in 25 fractions over 5 weeks, 2Gy per fraction). The research arm consists of the above plus cetuximab (400 mg/m^2 ^day 1, week 1 only, then 250 mg/m^2 ^weekly thereafter for a further 11 weeks).

During Phase II of the study, the trial will assess toxicity (unexpected and expected), activity and feasibility in 90 patients in the experimental arm. Activity will be measured as treatment failure-free rate and feasibility as the number of protocol dose modifications and delays. If toxicity, activity and feasibility are found to be unacceptable recruitment into the trial will stop. If the experimental arm is found to be active, safe and feasible, by the Independent Data Monitoring Committee (IDMC), recruitment will continue into Phase III of the study. Phase III of the study will recruit a further 120 patients into each arm (a total of 420 patients) and compare the overall survival of both groups. The experimental arm of CRT and cetuximab will be compared against the control arm of CRT alone. This second stage of the trial is powered to detect a survival advantage with the addition of cetuximab.

All patients randomised into Phase II will contribute to the Phase III comparison of overall survival. In addition to overall survival, Phase III of the study will also assess toxicity, HRQL, and cost effectiveness.

Detailed radiotherapy treatment protocols have been notably absent in recent UK Upper GI Cancer trials involving CRT, as have procedures to assess adherence to treatment recommendations. Therefore a detailed radiotherapy protocol and quality assurance procedure (Radiotherapy Trials Quality Assurance (RTTQA)) will be incorporated into this trial.

SCOPE1 has been ethically approved by the Research Ethics Committee for Wales and has approval from the Medicines and Health Care Product Regulatory Agency to be conducted in the UK. The Wales Cancer Trials Unit, a Cancer Research UK core funded and National Cancer Research Institute accredited Clinical Trials Unit, is coordinating the trial. Velindre NHS Trust is the sponsor for the trial. A Trial Steering Committee and an Independent Data Monitoring Committee has been set up to monitor the progress and safety of the study. The SCOPE1 Trial Management Group, including clinicians, clinical trial unit staff, patient representatives, nursing and pharmacy representatives, carry out the day-to-day running of the trial.

### Participant Eligibility

Eligible patients will have been diagnosed with carcinoma of the oesophagus, AC, SCC, or undifferentiated carcinoma, or Siewert Type 1 or 2 tumour of the gastro-oesophageal junction (GOJ) and selected for treatment with definitive CRT by an appropriate specialised MDT. Such patients may enter the trial if they meet all the necessary inclusion and none of the exclusion entry criteria (see Table 1).

**Table 1:** Inclusion and exclusion criteria for the SCOPE1 trial

Inclusion Criteria Patients meeting the following criteria can be included in the trial:
1. Histologically confirmed carcinoma of the oesophagus (adenocarcinoma or squamous cell or undifferentiated carcinoma) or Siewert Type 1 tumour of the gastro-oesophageal junction (GOJ) or Siewert Type 2 with no more than 2 cm mucosal extension into the stomach.
2. Age 18 or over
3. Have been selected to receive potentially curative definitive CRT by a specialist Upper GI MDT including a designated Upper GI surgeon.
4. Not suitable for surgery either for medical reasons or through patient choice.
5. Tumours staged with both endoscopic ultrasound (EUS) and spiral CT scan to be T1-4, N0-1 confirming localised, non-metastatic disease (both within 7 weeks prior to randomisation, but the most recent within 4 weeks). An attempted but failed or contra-indicated EUS is acceptable. Tumours should be staged according to the 6th edition of the American Joint Committee on Cancer's (AJCC) Cancer Staging Manual
6. Total disease length (primary and lymph nodes) less than or equal to 10 cm defined by EUS or CT if EUS attempted but failed or contra-indicated.
7. WHO Performance status 0-1
8. Adequate cardiovascular function for safe delivery of CRT in the opinion of the principal investigator
9. Adequate respiratory function for safe delivery of CRT in the opinion of the Principal Investigator
10. Adequate bone marrow and hepatic function (within 1 week prior to randomisation):
• Absolute neutrophil count (ANC) ≥ 1.5 × 10^9^/L
• White blood cell count ≥ 3 × 10^9^/L
• Platelets ≥ 100 × 10^9^/L
• Haemoglobin (Hb) ≥ 10 g/dL (patients' Hb should be corrected to > 10 g/dl before treatment)
• Adequate liver function (within 1 week prior to randomisation)
• Serum bilirubin ≤ 1.5× ULN
• ALT/AST ≤ 2.5× ULN
• ALP ≤ 3× ULN
11. Adequate renal function (within 1 week prior to randomisation): Glomerular filtration rate (GFR) assessed by EDTA clearance to be > 40 mL/min (or estimated by Cockcroft-Gault formula to be > 60 mL/min)
12. Patients who are fit to receive all protocol treatment.
13. Patients who are able and willing to administer capecitabine.
14. Patients who are of child bearing age are willing to use contraception.
15. Patients who have completed baseline quality of life questionnaires
16. Patients who have provided written informed consent prior to randomisation
**Exclusion Criteria** If any of the following criteria apply, patients cannot be included in the trial:
1. Patients who have had previous treatment for invasive oesophageal carcinoma or gastro-oesophageal junction carcinoma (not including PDT or laser therapy for high grade dysplasia/carcinoma in-situ).
2. Patients with metastatic disease i.e. M1a or M1b according to UICC TNM version 6.
3. Patients with any previous treatment for malignancy which will compromise ability to deliver definitive mediastinal CRT or may compromise survival (does not include patients with squamous cell carcinoma).
4. Patients who have had a previous malignancy during the previous 5 years
5. Patients with significant (> 2 cm) extension of tumour into the stomach
6. Patients with unstable angina or uncontrolled hypertension or cardiac failure or other clinically significant cardiac disease
7. Patients who have had major surgery or major trauma in the 4 weeks prior to randomisation.
8. Patients who have been treated with a monoclonal antibody in the 4 weeks prior to randomisation.
9. Patients who have been treated with radiotherapy in the 3 months prior to randomisation
10. Patients who need continued treatment with a contraindicated concomitant medication or therapy
11. Patients with known dihydropyrimidine dehydrogenase (DPD) deficiency
12. Patients with hearing impairment or sensory-motor neuropathy of WHO grade > 2
13. Women who are pregnant

Within 8 weeks prior to randomisation, a spiral/multislice CT scan (+/- PET) and an endoscopic ultrasound (to include recording of proximal and distal extent of primary tumour, location of lymphadenopathy and reference point for localisation on CT planning) should be performed. One of these assessments should be carried out within 4 weeks. Within 1 week prior to randomisation the following screening assessments should be performed: a history and physical examination (to include height, weight and WHO performance status); full blood count; serum renal, liver and bone profile (including serum magnesium); glomerular filtration rate; electrocardiogram; and pregnancy test in females of child bearing age.

### Sample Size Considerations

#### Phase II

The treatment failure-free rate in patients treated with CRT is approximately 60% at 24 weeks [35,42]. With the addition of cetuximab it is felt that a treatment failure-free rate of less than 60% would not be sufficiently large enough to warrant further investigation in a Phase III setting, but that a rate of 75% or higher would warrant further investigation. Using a Fleming's single stage design, p1 = 0.60 and p2 = 0.75, setting α = 0.05 and 90% power 83 patients would be required. To allow for 10% not being assessable and with a 1:1 allocation 90 patients will be recruited into each arm, a total of 180 patients. The IDMC will review the treatment failure-free rate, along with toxicity and feasibility, before they endorse continuation into Stage 2.

#### Phase III

Calculations were performed using the ART package [44] in Stata 9 assuming a 3 year recruitment period followed by 1 year of follow up (based on a 2-sided log rank test). The 2 year overall survival rate in patients treated with CRT is approximately 35%. In order to detect an improvement in 2 year overall survival from 35% to 47.5% (Hazard Ratio [HR] = 0.71) with 80% power at a 5% significance level a total of 420 patients (269 events) are required. A total of 210 patients will be recruited to each arm.

### Method of Randomisation

Patients are randomised centrally by the Wales Cancer Trials Unit using the method of minimisation which includes a random element. Patients are stratified for a number of clinically important stratification factors. The randomisation allocation ratio for control: research arm will be 1:1.

### Outcome Measures

#### Phase II

The primary outcome measure is treatment failure rate in the research arm. Treatment failure rate will be assessed at 24 weeks (12 weeks after completion of CRT) and is defined as pathologically confirmed (by endoscopic assessment and/or biopsy) residual disease and/or CT scan of thorax and abdomen showing progressive disease according to RECIST criteria. Treatment failure will also include progression or death due to disease before 24 weeks.

The secondary outcome measures are:

• **Toxicity: **will be scored using the NCI CTCAE v3.0 and RTOG late radiation morbidity scoring criteria at baseline, during treatment and at pre-specified time points on follow-up. Serious adverse events will be monitored "real-time" by the Chief Investigator and TMG.

• **Feasibility: **will be measured through the number of protocol dose modifications and delays.

#### Phase III

The primary outcome measure is overall survival (OS). Overall survival is calculated from the date of randomisation to death from any cause. Those patients still alive will be censored at the date last seen.

The secondary outcome measures are: toxicity (as above), HRQL, RTTQA and cost effectiveness:

• **HRQL**: This aspect of the trial aims determine whether cetuximab in addition to CRT:

◦ improves generic and disease specific aspects of HRQL following treatment than CRT alone. Specific HRQL domains that are expected to be better in the experimental group are: physical and role function, fatigue, dysphagia and eating restrictions

◦ is associated with poorer HRQL during treatment. Specific HRQL domains that are expected to be worse in the experimental arm include: dyspnoea, skin rashes and diarrhoea.

Generic domains of HRQL will be assessed with the EORTC core Quality of Life Questionnaire, the EORTC QLQ-C30[45]. This instrument has been well validated in many international clinical trials in oncology including oesophageal adenocarcinoma and squamous cell cancer. Disease specific and CRT associated symptoms and side effects will be assessed with the oesophageal cancer specific module, the EORTC QLQ-OES18 [46]. This has been validated and tested in patients receiving definitive CRT. The module includes scales assessing dysphagia, eating restrictions, reflux, dry mouth and problems with saliva and deglutition. The Dermatology Life Quality Index (DLQI) will also be administered [47]. This is a well validated, easy to use index which assesses the impact of dermatological conditions on patients' HRQL [48]. It has been included to accurately assess the impact of the acneiform eruption commonly seen with cetuximab. All patients who participate in the SCOPE1 study will complete questionnaires at: baseline, during and at end of treatment (Week 7 and week 13), post treatment (24 weeks, time of endoscopy and scan), 12, 18, 24 months and annually to 60 months.

• **Cost effectiveness**: A cost-utility analysis of cetuximab in addition to CRT in oesophageal cancer will be performed from the perspective of the UK National Health Service, but with consideration also given to patient and family related costs. The cost-effectiveness will be considered using two time horizons: within trial and lifetime. In order to undertake the latter a decision-analytic model will be developed to assess the costs and effects over the lifetime of patients. The model will be a Markov model and the transition probabilities will be based on parameter estimates derived from the trial (and other sources from within the literature). The within trial component will involve a comparison of the additional costs associated with the use of cetuximab in the treatment regimen ± any changes in resources utilised elsewhere. Costs of cetuximab will be based on discussions with clinicians and finance staff, while other healthcare resources utilised by patients during the study period will be collected via the healthcare resource utilisation log administered during treatment and at follow-up. At each of the 3-4 weekly visits, patients will be asked to indicate whether they have had any contacts with their GP, practice nurse, community nurse or attended hospital either as an outpatient or in-patient. They will be asked whether the contact was connected to their condition or for any other purpose. The involvement of others in providing transport and/or support will also be logged. In addition, they will be asked to indicate the medication they have been taking during the 3-4 week period. Consultations with healthcare professionals will be costed using published sources of unit costs and healthcare resources utilised will be added to the respective treatment costs in each arm. Quality adjusted life years (QALYs) will be derived from survival data generated during the second stage of the trial and from the EQ-5D scores collected at baseline, during treatment and at follow-up. A patient diary will also be used to collect additional data. These data will then be used to populate the lifetime model.

• **RTTQA**: A detailed QA program will be in place to ensure adherence to the protocol (see RTTQA section below). Major and minor deviations will be collected.

NB: Although activity, safety and feasibility will not be formally assessed in the control arm in Phase II, the randomisation of patients will continue using the same process and on a 1:1 allocation and therefore those patients in Phase II will be included in the analysis of Phase III.

### Data collection

Participants will be seen at hospital at randomisation, the end of each treatment cycle, then at Month 6, 9, 12, 16, 20, 24, 36, 48, and 60 after randomisation. Research staff at the hospitals will be expected to complete trial CRFs which record evidence of primary and secondary outcome measures.

### Statistical analysis

All analyses will be on an intention-to-treat basis, i.e. all patients randomised will be included, and all patients will be analysed according to their allocated group whatever treatment they received. Both Phase II and Phase III analyses will include:

• Descriptive statistics of the patient characteristics within each treatment group

• A CONSORT flow diagram of enrolment, intervention allocation, and follow-up

• Tables of toxicities at each timepoint (baseline, end of each treatment cycle, then Month 6, 9, 12, 16, 20, 24, 36, 48, 60)

• Treatment compliance during each cycle (in terms of proportions of patients with delay/reduction to CRT) within each treatment group. An exploration of predictors of poor compliance will be performed.

#### Phase II

When 6 month follow up data has been obtained for 83 patients in the research arm then the Stage 1 analysis will be performed. According to the Flemings design, we need to see 58 "failure free" patients out of 83 patients followed up to 6 months in the research arm.

#### Phase III

The main analysis will compare overall survival between the two groups using unadjusted logrank test. Final analysis will take place when 269 events (deaths) have been reported, which is expected to be approximately 1 year after accrual closes. The secondary outcome of proportions of patients with SAEs will be compared using a chi-squared test.

No formal subgroup analyses are planned to look at differences in primary and secondary endpoints between treatment groups within specific groupings based on patient characteristics. However, if any treatment difference is found we will look to see whether it is consistent across patient subgroups (defined by all pre-treatment factors collected) although this analysis will be exploratory in nature.

The HRQL data will be scored according to the algorithms described in the EORTC QLQ-C30 scoring manual [49]. All scales and single items are scored on categorical scales and linearly transformed to 0-100 scales where: (a) a high score for a symptom scale or item represents a high level of symptoms or problems, and (b) a high score for a functional scale represents a high or health level of functioning and a high score for the global health status/HRQL represents high QL. Groups of patients will be compared at agreed time points and overall for differences in these parameters. The treatment groups will be compared at the individual time points with appropriate adjustments being made for multiple comparisons. Because of the longitudinal nature of the data, an analysis that takes into account the repeated measures is also needed. A generalised linear modelling approach will be adopted. This will allow the appropriate error distribution to be used and will enable the analysis to take account of important prognostic factors.

The economic evaluation will assess the differences in mean costs between the trial arms and use with the differences in QALYs to generate a cost per QALY estimate. The EQ-5D allows estimation of quality adjusted life years (QALYs) which will be the main effectiveness measure in the economic analysis. Between group differences will be estimated using the area under the curve method adjusted for differences at baseline. Since cost data are often skewed, bootstrapping methods will be used to produce 95% confidence intervals alongside point estimates. A probabilistic sensitivity analysis will be undertaken and a cost-effectiveness acceptability curve produced. In addition, a series of one-way sensitivity analyses will be undertaken to assess the robustness of the estimate to changes in costs, resources utilised, utility scores and QALYs. In the case of non-dominance, results will be reported in the form of an incremental cost effectiveness ratio (ICER) which shows the extra cost of producing one extra QALY. A cost-effectiveness acceptability curve (CEAC) will be used to calculate 95% confidence intervals for the incremental cost effectiveness ratios.

### Radiotherapy Quality Assurance

The RTTQA for SCOPE1 consists of clinical oncologists, dosimetrists, trial management, medical physicist and research fellows. It is part of the NCRI RTTQA group.

An educational CD ROM is being distributed to all investigators. This CD ROM includes three example cases, each of which consists of a clinical summary, CT slice data, outlines and Radiotherapy plan information. GUINESS software is provided for viewing the CT, outline and planning data. A radiotherapy treatment planning and delivery protocol has been written and is supplied in hard copy and electronically on the CD ROM.

All clinical and radiation oncologists participating in the trial are required to outline a test case (CT data set for which is included on the educational CD ROM). All planning departments are required to plan this test case. This would usually be carried out on the outline completed by the Principal Investigator. A Plan Assessment Form (PAF) is completed that compares the planned test case with the delivery protocol for major and minor deviations. This, together with the completed plan, is assessed centrally by the SCOPE1 RTTQA team. All centres and Investigators must have a 'Pass' for the planned test case before entering patients into the trial.

On trial, centres are required to provide a PAF and a full dataset of images, structures, plan and doses, for each patient. The first clinical case from each investigator, a 10% sample and those with noted major deviations (on the PAF) from the protocol will be assessed centrally by the SCOPE1 RTTQA in real time.

## Discussion

This Cancer Research UK funded trial will define the most effective CRT regimen in terms of overall survival for patients with oesophageal cancer unsuitable for surgery due to the presence of co-morbidity or extent of disease. The Phase II/III design allows an early assessment of activity and toxicity of cetuximab in this context before progressing to the definitive Phase III endpoint. This provides an early stopping opportunity whilst also allowing trial momentum to be maintained. This efficiency saves years compared to running concurrent Phase II and Phase III trials by having one rather than two funding and regulatory approval applications and centre set up processes. This trial builds in health economic and HRQL measures which provide a well-rounded assessment of the impact of the experimental regimen. It also has a RTTQA component which standardises radiotherapy delivery within the trial.

## Competing interests

SWG has received research funding from Roche and Merck. Otherwise none of the authors have any competing interests apart from receiving free drug from Merck for this trial.

## Authors' contributions

TDC was responsible for the research question. GOG, TDC, IG, SG, JNS, CH, LSN were responsible for the design of the trial and contributed to the writing of the study protocol. GOG is the Scientific Director and TSM the Clinical Director of the Wales Cancer trials Unit and has overall responsibility for running the trial at the WCTU. RAM is the Trial Manager for the trial. CJP is the Health Economist for the trial and participated in the study design. JMB designed the HRQL aspects of the study. All authors have read and approved the final manuscript.

## Pre-publication history

The pre-publication history for this paper can be accessed here:

http://www.biomedcentral.com/1471-2407/11/466/prepub
